# Regulatory Mechanisms of Plant Growth-Promoting Rhizobacteria and Plant Nutrition against Abiotic Stresses in Brassicaceae Family

**DOI:** 10.3390/life13010211

**Published:** 2023-01-11

**Authors:** Arshad Jalal, Carlos Eduardo da Silva Oliveira, Fernando Shintate Galindo, Poliana Aparecida Leonel Rosa, Isabela Martins Bueno Gato, Bruno Horschut de Lima, Marcelo Carvalho Minhoto Teixeira Filho

**Affiliations:** 1 Department of Plant Health, Rural Engineering, and Soils, Campus of Ilha Solteira, São Paulo State University (UNESP), Av. Brasil, 56- Centro, Ilha Solteira 15385-000, SP, Brazil; 2 Faculty of Agricultural and Technological Sciences, Campus of Dracena, São Paulo State University (UNESP), Dracena 17900-000, SP, Brazil

**Keywords:** microorganisms, stressful conditions, sustainability, abiotic stresses, nutrition, Brassicaceae

## Abstract

Extreme environmental conditions, such as abiotic stresses (drought, salinity, heat, chilling and intense light), offer great opportunities to study how different microorganisms and plant nutrition can influence plant growth and development. The intervention of biological agents such as plant growth-promoting rhizobacteria (PGPRs) coupled with proper plant nutrition can improve the agricultural importance of different plant species. Brassicaceae (Cruciferae) belongs to the monophyletic taxon and consists of around 338 genera and 3709 species worldwide. Brassicaceae is composed of several important species of economical, ornamental and food crops (vegetables, cooking oils, forage, condiments and industrial species). Sustainable production of Brassicas plants has been compromised over the years due to several abiotic stresses and the unbalanced utilization of chemical fertilizers and uncertified chemicals that ultimately affect the environment and human health. This chapter summarized the influence of PGPRs and nutrient management in the Brassicaceae family against abiotic stresses. The use of PGPRs contributed to combating climate-induced change/abiotic factors such as drought, soil and water salinization and heavy metal contamination that limits the general performance of plants. Brassica is widely utilized as an oil and vegetable crop and is harshly affected by abiotic stresses. Therefore, the use of PGPRs along with proper mineral nutrients management is a possible strategy to cope with abiotic stresses by improving biochemical, physiological and growth attributes and the production of brassica in an eco-friendly environment.

## 1. Introduction

Brassica is one of the most important and economical vegetables of the Brassicaceae family [1] and includes several species (*Brassica oleracea*, *Brassica rapa*, *Nasturtium officinale*, *Raphanus sativus*, *Diplotaxis tenuifolia* and *Eruca vesicaria*), containing secondary metabolites and beneficial contents of putative health-promoting compounds [2]. Brassicaceae are a rich source of primary and secondary metabolites (amino acids, sugars, indoles, phenolics and glucosinolates) that help in the production of antioxidants [3,4] to promote tolerance to biotic and abiotic stresses [5]. Brassicaceae are emergently adapting as a research model crop in plant science due to their interaction with biotic and abiotic stresses as their high defensive mechanisms and a series of alterations in metabolites allow them to survive under climatic extremes [6]. Therefore, proper management practices are needed when encountering extreme environmental conditions (drought, salinity, temperature, heavy metals and nutrients deficiency) and to ensure optimal plant growth and productivity [7].

Abiotic stresses disturb plant physiology and metabolism, which leads to the reduction of plant growth and productivity [8]. The growth, yield and quality of Brassica grown in arid and semi-arid areas were extremely affected by drought conditions [9]. In addition, nutrient limitation is another vulnerable condition that alters plant growth, production and quality. Plants adapt different physiological and biochemical functions to adjust to extreme challenges and avoid injuries under abiotic stresses [10]. Macronutrients mobilize and assimilate along with organic compounds that could improve plant growth and development and mitigate plant abiotic stresses [11]. The absorption of chromium (Cr), zinc (Zn), iron (Fe) and manganese (Mn) was increased with chelating agents of low molecular weight, which led to the improvement of oil content in *Brassica juncea* up to 35% [12]. The imbalanced utilization of macro and micronutrients may cause metal toxicity in several crop plants [13]. However, Brassica species deal with the hyper-accumulation of these nutrients by improving biochemical processes and the mobilization of nutrients through the roots–shoot system [14]. In addition, the root rhizosphere is influenced by different biotic and abiotic factors including soil and root type and plant species and age. Hence, plant growth-promoting rhizobacteria (PGPR) are classified into several groups on the basis of their capacities and taxonomical status. These bacteria activate several mechanisms that alter soil organic matter to an instantly available form [15], as well as the regularization and transformation of soluble sugars, proline, amino acids and mineral nutrients in the soil above plant parts, thus improving nutrient accumulation in nutrient-deficient soils [16].

The plant and bacteria association promotes nutrient uptake and assimilation, which favors the plants’ tolerance to biotic and abiotic stresses [17]. Plants and microbial communities are the components of similar limited resources with a different relationship. However, plants assist microbial communities with available nutrients from the soil rhizosphere [18] and improve nitrogen mineralization, which can enhance the uptake of other nutrients for a higher performance and yield of plants [19]. The positive association (symbiosis) and negative association (pathogenesis) of the plant rhizosphere microbial community can affect nutrient availability and resource partition, thus increasing or reducing crop production, respectively [18,20]. The positive association of the microbial community increases their activities in the rhizosphere of host plants, which can improve the soil organic matter (SOM) content and nutritional status of the plant [21]. Beneficial bacteria are the first soil-borne communities that alter and re-adjust in stressful environments for their survival; however, their activities and configurations are the first affected factors under stress [22]. The plant growth-promoting rhizobacteria community is vulnerable to stressful conditions of low water potential and nutrient availability that may be reflected in the form of physiological stress in the plants [23].

The eco-physiological and functional activities of nutrients and PGPRs need proper attention and extensive research to improve plant tolerance to abiotic stresses. Therefore, this review highlighted the interaction between plant growth-promoting rhizobacteria and mineral nutrition and their influence on the tolerance to abiotic stresses in the Brassicas plant species.

## 2. Adverse Effects of Abiotic Stress in Plants

Abiotic stresses are the foremost confining factors for agricultural productivity. Crop plants overcome the drastic external pressure of intrinsic mechanisms caused by environmental and edaphic conditions that affect the growth, development and productivity of plants [24,25]. The sustainable production of vegetables such as Brassicas around the world has been compromised due to several harsh environmental conditions and the unbalanced use of synthetic fertilizers and uncertified chemicals over the years that affect the environment and human health and led to inadequate climatic conditions. Abiotic stresses consist of drought, low/high temperature, salinity, light intensity, flooding, heavy metals toxicity and nutrient starvation. The extensive use of chemicals, macro and micronutrients, non-essential elements and radionuclides are the main sources of metal toxicity in soil [13,25]. Brassicaceae are capable plant species that deal with the hyper-accumulation of heavy metals through their biochemical expression, acquisition and re-mobilization in roots [13,14]. Waterlogging/flooding is an excess of soil water that can reduce oxygen availability in plant root systems and thus negatively affect crop growth and yield [26]. Flooding has negatively affected lipid biosynthesis and the yield of several rapeseed varieties [27].

**Figure 1:**
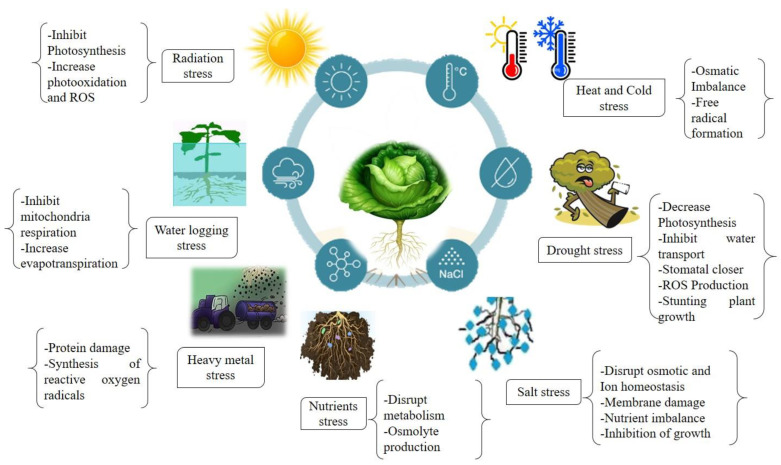
Effects of abiotic stresses and their consequences on Brassicaceae.

Cold stress is associated with chilly weather (0–15 °C) and frosty weather (<0 °C) that leads to the disturbance of the photosynthetic process and reduces the primary production of *B. oleracea* [28]. Cold stress impairs metabolic and enzymatic activities that can disrupt the cell membrane and cause seed rotting in Brassica plants [29,30]. Light radiation (low or high) affects plant morphology and the root–shoot ratio [31]. Exposure of broccoli (*B. oleracea*) to ultraviolet (UV) light can increase ascorbic acid [32,33]. High light causes photoinhibition of the photosystem and protein degradation in *B. rapa* plants [34]. In short, abiotic stresses alter several internal functions of plants by disturbing homeostasis, physio-biochemical and molecular attributes, such as water and nutrient use efficiency and assimilation, osmotic adjustment, disruption of membrane integrity and enzymatic activities, as well as reduction in photosynthetic efficiency [29,31,34]. The abiotic stresses and their consequences are summarized in Figure 1.

## 3. Use of Plant Growth-Promoting Rhizobacteria to Mitigate Adverse Effects of Abiotic Stress

In recent years, the contribution of rhizosphere microorganisms to increasing plant growth and crop productivity as well as tolerance to biotic and abiotic stresses without causing pathogenicity have been discussed in the literature [35]. Several genera of plant growth-promoting rhizobacteria (PGPR) including *Azospirillum*, *Bacillus*, *Rhizobium*, *Pseudomonas* and *Bradyrhizobium* showed positive interactions with different vegetables species [36,37]. Several previous studies highlighted the capacity of different PGPRs in biological nitrogen fixation (N_2_) [38,39], increasing the availability of iron (Fe) [40], phosphorus (P) and zinc (Zn) solubilization and transportation [41,42]. The PGPRs also improved the performance and growth of plants through the production of phytohormones such as gibberellins, ethylene, cytokinin, auxins and salicylic acid [43,44].

The use of PGPRs has contributed to combating climate-induced changes (abiotic factors) such as uneven rainfall (drought), soil and water salinization and heavy metal contamination that limit the general performance of plants [44,45]. These microorganisms improve soil fertility and structure, which contribute to a successful adaptation of the plant under stressful conditions [45]. Researchers have been focused on the use of these microorganisms with emphasis on bacteria of the genera *Azospirillum*, *Bacillus*, *Pseudomonas*, *Rhizobium*, *Bradyrhizobium*, *Herbaspirillum* and *Burkholderia* [36,38].

**Figure 2:**
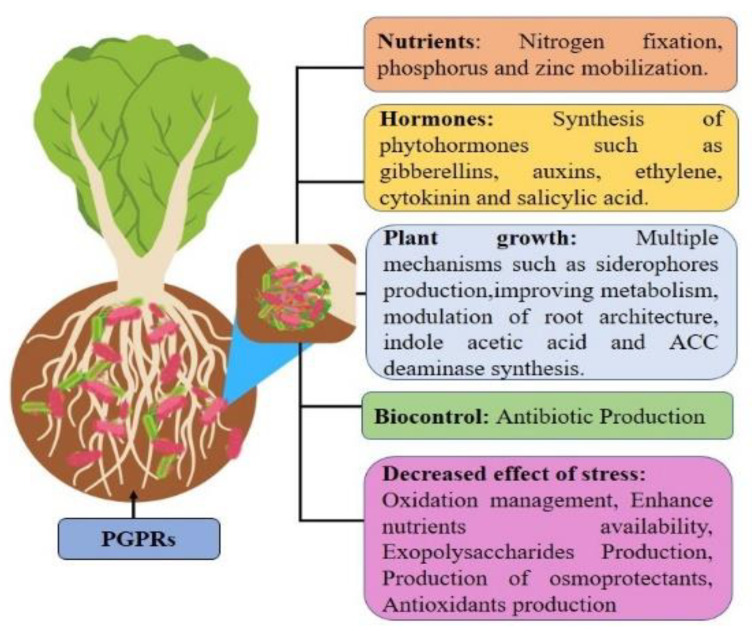
Role of plant growth-promoting rhizobacteria in Brassica species against abiotic stresses.

PGPRs exist in the rhizosphere and tissues of plants, which may adapt multiple mechanisms including the synthesis and exudation of phytohormones (indole-3-acetic acid (AIA)), cytokinin, ethylene and gibberellins [46]; synthesis of plant growth-regulators including nitric oxide [47]; abscisic acid [48]; polyamines such as spermidine and spermine [49]; increase solubilization and availability of nutrients [50,51]; increase nitrate reductase activity and nutrient use efficiency [38,52]; biocontrol of phytopathogens and diseases [53]; and protection of plants against water and saline stress and toxic chemical elements of the soil [54]. In addition to assisting in biological nitrogen fixation, PGPRs have the ability to enhance cell membrane stability of the leaf and reduce the rate of leaf abscission during drought stress conditions [55]. Several PGPRs improve the tolerance capability of plants by producing certain phytohormones [56] that can be used for heavy metal remediation, mobilization or immobilization from soil into plant tissues [57,58]. These microbes also utilized 1-aminocyclopropane-1-carboxylic acid (ACC) to prevent ethylene production [59] and mitigate stresses by endophytic biota, which were caused due to high radiation and light stress [60]. Plant growth-promoting rhizobacteria adapted several mechanisms to improve the growth and development of the plants of the Brassicaceae family under abiotic stresses (Figure 2).

Plant growth-promoting rhizobacteria (PGPR) promote plant tolerance to abiotic stresses through the adaptation of several mechanisms as well as down- or up-regulating stress genes [61]. The inoculation of rapeseed plants with *Pseudomonas* sp. and *Azospirillum* sp. mitigate salt stress [62] by increasing the solubilization and availability of macro- and micronutrients for better uptake in the above-ground part of the host plant [63,64]. PGPRs prominently improved root–shoot fresh and dry weights, leaf area, chlorophyll and several growth-promoting hormones, which ultimately improved the seedling growth of *B. oleracea* and *B. napus* [65,66]. Flooding is another abiotic stress that harshly reduces antioxidant activities; however, inoculation with bio-fertilizers (*Azotobacter chroococcum*, *Azospirillum* spp. and *Pseudomonas* spp. and *Azospirillum* spp., *Pseudomonas fluorescens* and *Basillus subtilis*) via seeds and foliar efficiently alleviate flooding affects in canola by increasing growth and yield [67]. In this context, the supply of these rhizobacteria or PGPRs to plants of the Brassicaceae family brought benefits to their cultivation in abiotic conditions (Table 1).

## 4. Plant Nutrition to Mitigate Adverse Effects of Abiotic Stress on Brassicas

Plants develop extensive adaptive and/or resistance mechanisms to sustain productivity and survival under stressful conditions. However, adequate nutrient application is an imperative tool to meet the Sustainable Development Goals to attain food and nutritious security and promote sustainable productivity under climate extremes [79]. Optimization of nutrient content (macro- and micronutrients, secondary nutrients and heavy metals) in soil and plant systems have been reported to enhance crop adaptation to resilience conditions, as these are structural elements of several co-factors and enzymes. Nutrients assist structures’ stability of protein and alleviate reactive oxygen species (ROS) production. The versatility of nutrient application under severe environmental conditions has significantly improved the yield and quality traits of various crops [80].

**Figure 3:**
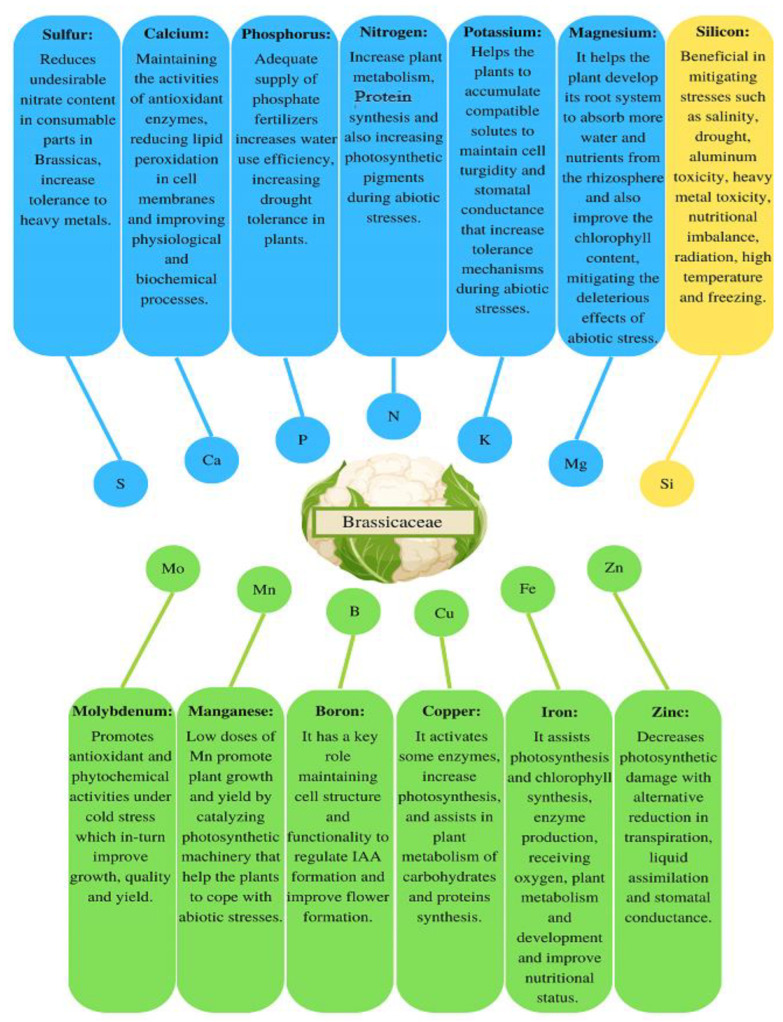
Effect of macronutrients (indicated in blue color), silicon (orange color) and micronutrients (green color) on different functions of Brassicaceae crops.

Fertilizers are considered the most important and crucial inputs to achieve greater crop growth and production in modern agriculture [81]. Plants require NPK and other essential micronutrients such as iron (Fe), copper (Cu), zinc (Zn), manganese (Mn), molybdenum (Mo), nickel (Ni), chlorine (Cl) and boron (B) in very small quantities for better performance and yield. These elements are collectively considered as essential for humans and animals and their deficiency can affect their metabolic, physical and mental development. Macro- and micronutrients play a critical role in the effectiveness of several biological compounds and enzymes for the proper functioning of different metabolic processes. The relevance of macronutrients’ essentiality for higher yield and nutritional status has been increasing over several decades [82]. Ensuring that plants are well-fed with essential nutrients is a cost-effective strategy with the capacity to mitigate abiotic stresses and enhance productivity [79,81]. The effect of macro- and micronutrients on different functions of Brassicaceae crops promotes plant growth and increases tolerance to abiotic stresses (Figure 3).

### 4.1. Macronutrients

Macronutrients are considered to be significant drivers for enhancing the yield and quality parameters of crop plants. Traditional fertilizer application in a field may not fulfill the demands of individual plants while over and/or under application causes soil quality degradation, groundwater pollution and reduction in productivity. Leaf nutrition of rapeseeds is an important factor to optimize fertilization and productivity, alongside contributing to commercial and environmental profits [83]. Better management of macronutrient fertilizers can improve plant growth and yield under stressful conditions. The nutrients and their functions in the crop plants are discussed below in detail.

#### 4.1.1. Nitrogen

Nitrogen (N) is the most needed nutrient for most cultivated plants, and it directly affects plant development and yield [84,85,86,87,88,89]. Nitrogen is the main constituent of the atmosphere, but its availability is still one of the main limiting factors for the productivity of terrestrial ecosystems including agro-ecosystems [90]. Nitrogen plays an important role in plant nutrition and development [87], such as the synthesis and production of phytohormones, co-enzymes, nucleic acids, secondary metabolites, chlorophyll and proteins content [91].

Several studies have reported that N fertilization promoted different species of Brassicaceae including oilseed producer crops such as rapeseed (*B. napus*) [92,93], brown mustard (*B. juncea*) [94,95] and turnip rape (*B. rapa*) [96] and horticultural crops such as radish (*Raphanus sativus*) [97], cauliflower (*B. oleracea* L. var. botrytis) [98,99], cabbage (*B. oleracea* L. var. Capitata) [100,101], broccoli (*B. oleracea* L. (var. italica) [102,103], kale (*B. oleracea* L. var. sabellica) [104,105] and arugula (*Eruca vesicaria* subsp. Sativa) [106].

Abiotic stress conditions alter the N metabolism of Brassicaceae plants [94], negatively affecting N uptake and assimilation, N use efficiency (NUE), photosynthetic capacity and plant growth [107], particularly under prolonged (24 h) stress exposure [108]. The interaction of N fertilization and abiotic conditions plays an important role in determining the potential of plant development and abiotic stress tolerance. Stress relief depends on the type of N fertilization; applying ammonium (NH_4_^+^) to plants resulted in a stronger tolerance to heat stress as compared to the fertilization with nitrate (NO_3_^−^) [109]. In addition, N fertilization can compensate for the negative effects of abiotic conditions by facilitating carbon partitioning, cell membrane stability, osmoregulation and antioxidative mechanisms that could improve plant growth and development as well reduce leaf senescence under extreme environmental conditions [110].

#### 4.1.2. Phosphorus

Phosphorus (P) is a primary macronutrient with a structural function in plants. It is involved in drivers of metabolic functions including respiration, energy storage and transportation, production of nucleic acid, membrane stability, catalyze enzymes activities, redox reactions and contribution to carbohydrate metabolism [111]. As with other plant families, P is one of the important nutrients for the Brassicaceae family that directly affects its development and productivity [112]. Holzschuh et al. [113] studied different doses of P fertilizer in Brassicas and reported that the species of this family are highly demanding of P availability in the soil, especially broccoli (*B. oleracea* var. itálica) and cauliflower (*B. oleracea* var. botrytis). The optimal management of P fertilization in vegetables is essential for their proper growth, development and yield [112]. Phosphorus deficiency in soil and plants directly affects vegetable vigor, establishment and root development, thus disrupting water use efficiency [114]. Several plants of the Brassicas species have the capability to tolerate and respond to various types of stresses through hormonal stimulation, ion exchange, antioxidant enzymes and the activation of signaling flow in their metabolic and genetic boundaries that mitigate stressed conditions [115].

Application and management of appropriate P fertilization has increased water use efficiency against drought stress [116,117]. Jones et al. [118] indicated that adequate soil P contents compensate for the impact of drought stress on the growth and yield of plants. Application of P source fertilizers may reduce the drastic effects of water scarcity during pollen formation or the reproductive stage that could increase flower and pod production, resulting in a greater yield and high protein content in grains [119]. Phosphate fertilizers improved the performance of *B. juncea* under salt stress by increasing plant dry mass and P uptake while lowering the Na^+^/K^+^ ratio [114]. Phosphorus fertilization adapts different mechanisms that immobilize the metal content in soil [120] by reducing their dissolution under the low pH range of soil, hence leading to the reduction of the bioavailability and uptake of metals by plants [121]. Phosphate fertilization increases the pH of soil solution to constrain absorption of heavy metals, as their availability decreases with increasing P fertilization [122].

#### 4.1.3. Potassium

Plants develop a wide range of adaptive and resistive strategies that sustain productivity and survival under stressful conditions. Plant tissues may adjust osmotic potential through the absorption of various compatible osmolytes such as inorganic ions, carbohydrates, organic acids and free amino acids [123,124]. Plants adjust osmotic potential by regulating stomatal conductance, photosynthesis, leaf turgidity and plant growth rate under drought, salt and high-temperature stresses through potassium (K) osmolytes [125]. Potassium is one of the major inorganic osmolytes that enable osmotic regulation and adjustment during stress conditions. Potassium ion absorption protects plants from harmful impacts of different stresses including drought, salinity, metal toxicity and high or cold temperatures by osmotic adjustment and maintenance of stomatal conductance, protecting cell integrity and increasing photosynthesis as well as via the detoxification of reactive oxygen species [123].

In addition, K is a crucial element for the distribution of photo-assimilates in root systems [126] that protects plants against most abiotic stresses including metal toxicity such as Cd-induced oxidative damage [127], Zn toxicity [128], NaCl toxicity [129], drought stress [130] and high radiance incidence [131]. Potassium supplementation increases the adjustment of stomata, which regulates carbohydrate formation and the growth of *Nicotiana rustica* during stress conditions [125]. Samar-Raza et al. [132] reported that application of K fertilizer under drought stress enhanced the tolerance of wheat (*Triticum aestivum* L.) by reducing toxic elements’ absorption and enhancing physiological efficiency and yield [133].

#### 4.1.4. Calcium

Calcium (Ca) plays a vital role in the physiological functions of plants and acts as a second messenger element of external signals for the higher performance of plants. It has an essential role in the structure and stabilization of the cell wall and membrane, regulating metabolic, enzymatic and hormonal processes [134]. The alteration in free cytosolic Ca^2+^ ion contents is validated during naturally occurring abiotic stimulants (low and high temperature/light, tensions, high osmotic and oxidative tensions, also during biotic stimulants (nodulation aspects and fungal drivers)) [135]. It also has an explicit function in the performance and maintenance of plant development and detoxification of heavy metals [136]. The main function of Ca^+^ ions under heavy metal stress is to maintain the activities of antioxidant enzymes, reducing the peroxidation of lipids in the cell membrane and improving the physio-biochemical processes of plants [127,137].

#### 4.1.5. Magnesium

Magnesium (Mg^2+^) is an essential nutrient for plant growth [138], regulating cell membrane stability, carbon fixation, chlorophyll synthesis, carbohydrate transport, enzymatic activities and reproductive process [139,140,141]; thus, it helps plants to adapt defensive mechanisms against abiotic stresses [142]. Plants under Mg nutrition improve root growth and root surface area that increase water and nutrient uptake from the rhizosphere and enhance transportation of photo-assimilates and carbohydrate synthesis, which can mitigate drought-stress-induced deleterious changes [143]. Magnesium transports carbohydrates from roots to shoots and helps in the fixation of photosynthetic CO_2_ during the reproductive growth stage under salt stress. The efficiency of Mg foliar fertilization is right-away associated with the distribution of nutrients within plants [144]. Nutrient solution with Mg fertilization improved the shoot growth of *B. rapa* L. var. pervirdis under cadmium (Cd) toxicity [145].

Deficiency of Mg is one of the common nutritional syndromes in plants, which may have drastic impacts on agricultural productivity and quality [146] and lead to morphological and physiological abnormalities of plants [147]. Plants produce antioxidants and antioxidative defensive enzyme activities, especially ascorbic acid during the stress of Mg deficiency [148]. The glutathione-producing ascorbate-determined H_2_O_2_ scavengers are responsible for ascorbic acid that can enable the plants to detoxify ROS production to protect plants from climate extremes [149]. Glutathione homeostasis can be regulated through the over-production of glyoxalase genes that can help the plants to sustain Mg content during stressful conditions and increase tolerance to metalliferous soil [150,151].

Magnesium transporters are also involved in metal transport. Under the low Mg content, nickel (Ni^+^) is well-cited for the suppression of electron flow and impairing photosynthesis functions by replacing Mg^2+^ in chlorophyll fragments. Adequate fertilizer of Mg alleviates the Ni^+^ effect in the root rhizosphere that may reduce the negative probability of Ni at the outer surface of the plasma membrane by replacing the targeted ionic binding site [152]. The Mg transporter (AtMHX) from Arabidopsis acts as an H^+^ exchanger with Zn and Mg and is confined to the vacuole membrane [153]. The AtMGT1 protein derived from AtMGT (transporter gene of Arabidopsis) family in the plasma membrane exhibited greater attraction to Mg^2+^ ion, which helped in the re-distribution of Ni^+^, Ca^2+^, Fe^3+^, Mn^2+^ and Cu^2+^, when they are present in high concentrations [154].

#### 4.1.6. Sulfur

Sulfur (S) is among the very active macronutrients in plant metabolism, which is why it is recognized alongside nitrogen (N) as a key nutrient for plant development [155]. Sulfur is used by plants to assimilate with a variety of organic compounds that are essential for the growth, development and mitigation of plant stress [11,156]. It is also responsible for making vegetables softer and adding greater commercial value [157]. Sulfur is predominantly found in the soil and is one of the main nutrients that is absorbed by plants in the form of sulfate anion (SO_4_^2−^) from organic matter and a small proportion from the atmosphere in the form of sulfuric gas [158].

Kohlrabi (*B. oleracea* L.) is one of the crucially demanding S vegetables of the Brassicas family, which absorbs 1.5 kg S ton^−1^ of yield. Sulfur deficiency can inhibit leaf formation and change young leaves’ color from dark green to light green or yellowish. Proper S fertilization in kohlrabi (*B. oleracea* L.) improves tuber yield and reduces the undesirable nitrate content in consumable parts [159]. Canola (*B. napus* L. var. Oleifera) is also one of the most demanding S vegetables in reproductive phases as compared to other winter crops, as it exports a large amount of S to the grains [160]. Sulfur is one of the known nutrients that performs an imperative role in the tolerance to heavy metal toxicity [161].

Chromium is actively transported across the plasma membrane and appears to be mediated by transporters, which are primarily responsible for sulphate uptake [162,163]. This suggests the action of this molecule inhibit the absorption of heavy metals that are toxic to plants (Table 2).

### 4.2. Silicon

Silicon (Si) is the second most abundant chemical element after oxygen in the earth’s crust [199,200]; however, it is still not available directly to plants and is commonly adsorbed with oxides and silicates, affecting plant nutritional status [180,201,202,203]. In addition, the low dissolution of Si in the soil decreases its availability; thus, it occurs in a very low amount [204].

Plants uptake Si mainly from dissoluble mono-silicic acid (H_2_SiO_4_), a noncharged molecule which plays a significant role to increase plant resistance to abiotic and biotic conditions [205,206,207]. Silicon is distributed via xylem in the form of hydrated amorphous silica/silica bodies (SiO_2_.nH_2_O) and pledged to the epidermis of cell membrane. After deposition to the cell membrane, Si is no longer available for further distribution into the above-soil parts of the plants [208]. The transport of H_4_SiO_4_ occurs in a similar direction to transpiration (mass flow). Therefore, drought conditions increase the deposition of Si in the regions of leaf epidermis to protect water from high transpiration [209].

All soil-grown plants had Si constituents ranging from 0.1 to 10% of dry weight of plants [180,210]. However, Si is classified as a beneficial element, with it being an imperative element for several crops, specifically rice (*Oryza sativa* L.) and sugarcane (*Saccharum officinarum* L.). Moreover, its role has been well documented for the performance, growth and development of different Gramineae family crops [180,206,207,211,212,213]. This chemical element has been reported to be beneficial in mitigating abiotic stresses including heavy metal toxicity, salinity, high temperature, drought, radiation, aluminum toxicity, lodging, nutrient imbalance, wounding and freezing [214,215]. Rapeseed is one of the most studied plants of the Brassicaceae family regarding Si application to alleviate abiotic stress conditions [211], with the most common improvements reported in plant resistance to cold stress conditions, as well as the formation of larger seeds [216]. Table 2 summarizes the studies with Si fertilization in Brassicaceae plants under abiotic stress in the last decade (2004–2020).

### 4.3. Micronutrients

Micronutrients (zinc (Zn), iron (Fe), manganese (Mn), molybdenum (Mo), boron (B), copper (Cu) and chlorine (Cl)) improve plant health, water use efficiency, biomass production and provide systemic response against abiotic stresses [217,218,219]. Whereas plant growth-promoting rhizobacteria (PGPR) promote plant growth and tolerance to abiotic stresses by adapting and altering certain mechanisms, the production of ACC (1-aminocyclopropane-1-carboxylate) deaminase reduces ethylene synthesis, as well as alters phytohormones and antioxidative enzymes synthesis, and improves nutrient uptake [115,220].

Micronutrients may influence directly or indirectly the stress affecting plants due to their role in several enzymatic and metabolic activities [221]. Abiotic stress such as drought harshly impairs mineral nutrient translocation from soil to plant parts [222,223]. The Brassicaceae family is one of the most nutrient-demanding plant species, which is highly affected by inadequate nutrients application [224]. Therefore, deficiency of micronutrients disrupts the net-assimilation rate and stomatal conductance, electron transportation in photosynthesis, chlorophyll content, root–shoot ratio and antioxidant activities of cabbages, turnip and canola under abiotic stresses [225,226,227,228,229,230,231]. Salinity is a critical challenge to high production, physiological and biochemical attributes and nutrient uptake in Brassicaceae species [232]. Brassicas adapted certain mechanisms and variations, especially physiological variations to cope with salinity [233]. Salinization in plant systems can be ameliorated with foliar nutrient spray and rhizosphere micronutrient availability and uptake [234]. The accumulation of sodium (Na^+^) and chlorine (Cl^−^) ions increases osmotic potential and decreases water availability and nutrient uptake through plant roots [235].

Several studies regarding the Brassicaceae family indicated that most of the species grown on contaminated soils with high accumulation of nutrients (Zn and Cu) and non-essential metals (Pd, Cd, Ni and Cr) [236,237,238,239]. Plants of *B. juncea* have the ability to accumulate high amounts of Cd, Cu, Ni, Cr, Zn, Fe, Co, Pb and Se from metal-contaminated sites [240,241,242]. Rapeseed subjected to early waterlogging stress resulted in higher accumulation of Mn, Fe, Zn and Cu in the leaves and caused toxicity [243]. Zinc is one of the efficient nutrients in the reduction of heat stroke by improving biochemical activities and superoxide dismutase (SOD) content in *B. rapa* [244,245]. Boron and Mn application in winter rapeseed (*B. napus*) positively influenced pod production, photosynthetic rate, N-metabolism, antioxidant activities and improved N and Ca contents in seeds [227,246]. High UV-B radiation may alter nutritional status, disturb plant cell metabolism, increase pathogens and disease tolerance [247], whereas light-emitting diodes (short duration blue light) enhanced phytochemical activities and micronutrient (Zn, Mn, Mo, B, Na, Fe and Cu) concentration in Broccoli (*B. oleacea* var. italica) [248,249] (Table 3).

## 5. Conclusions

Based on the updated literature, this review highlighted the importance of adequate and balanced nutrition against abiotic stresses in Brassicas species to ensure food and nutritious security. Proper management of macronutrients, micronutrients and silicon under certain conditions of abiotic stress could improve nutritional and physiological status, thus resulting in higher productivity and quality of Brassicas plants. Balanced application of macro- and micronutrients mitigates abiotic-stress-induced changes in Brassicas plant species by stimulating absorption and accumulation mechanisms for better survival.

The use of plant growth-promoting rhizobacteria (PGPR) has a critical role in combating climate-induced changes such as uneven rainfall (drought), soil and water salinization and heavy metal contamination, which limit the general performance of Brassicas plant species. Among the PGPRs, the genera *Azospirillum*, *Bacillus*, *Pseudomonas*, *Herbaspirillum* and *Burkholderia* are well studied for increasing plant nutrition, tolerance to pathogens and climate extreme conditions, and hence could improve plant performance and productivity in adverse growing conditions. Therefore, inoculation with PGPRs can increase productivity of Brassicas grown under abiotic stress conditions.

In the future, attention needs to be paid to the response of Mg and micronutrient application on crop resilience under different abiotic stresses. Dose-response management and multiple interactions of nutrients and heavy metals still need further investigation. Bio-fortification via foliar spray of micronutrients is a cost-effective strategy in alleviating global food and nutritious security which requires future advances and intensified research. The intervention of nano-fertilizers on the basis of integrated evidence is required to reduce the gap. The expansion of enhanced detection, tracking and monitoring strategies may be the best early detection technique for abiotic stresses which can also control yield losses and lethal impacts on the nutritional security of crops.

## Figures and Tables

**Table 1:** Summary of the positive effects of PGPR in mitigating unfavorable abiotic stress conditions in Brassicas (2008–2020).

Crops	Abiotic Stresses	Positive Effect of PGPR	Reference
Radish	Salinity	*Bacillus subtilis*, *B. atrophaeus* and *B. spharicus* reduced osmotic effects of salinity to improve production.	[68]
Radish	Salinity	*Bacillus subtilis* and *Pseudomonas fluorescens* improved morphological and biochemical attributes as well as hormonal levels of plants.	[69]
Rapeseed	Drought	Inoculation of rapeseeds with *Pseudomonas fluorescens* or *P. putida* improved yield, 1000-grain weight, grains/pod, pods and branches/plant.	[70]
Rapeseed	Heavy metals	Use of *Bacillus megaterium* reduced soil Ni concentrations through the activity of IAA and solubilization of P.	[71]
Rapeseed	Salinity	*Pseudomonas* sp. and *Azospirillum brasilense* mitigated harmful effects of salinity by increasing leaf water content, activity of antioxidant enzymes, leaf area, osmolyte production, productivity and leaf nutrient concentrations.	[72]
Rapeseed	Heavy metals	Use of *Pseudomonas* sp. A3R3 and *Psychrobacter* sp. SRS8 reduced Zn toxicity in the soil due to the production of hormones and siderophore activity.	[73]
Rapeseed	Heavy metals	Use of *Arthrobacter* sp., *Bacillus altitudinis* SrN9, *B. megatherium* reduced soils cadmium contamination by producing IAA and siderophores.	[64]
Cabbage	Salinity	*Azotobacter chroococcum* minimized salt stress by increasing root development and IAA.	[74]
Cabbage	Drought	Inoculation with *Bacillus megaterium*, *Pantoea agglomerans* and *Brevibacillus choshiensis* improved physiology of membrane integrity and increased accumulation of osmolytes, antioxidant enzymes, hormonal production, decreased electrolyte leakage and production of ROS-eliminating enzymes	[75]
Canola	Salinity	*E. cloacae* improved tolerance to saline soils by promoting root–shoot growth and increasing production of phytohormones.	[76]
Turnip	Heavy metals	*B. megaterium* reduced soil contamination with cadmium and lead by the synthesis of IAA and siderophore activity.	[77]
Turnip	Drought and phytotoxicity of Zn and Cu	Inoculation with *Pseudomonas libanensis* TR1 and *Pseudomonas reactans* Ph3R3 reduced phytoremediation of metals polluted soils and increased relative water content by the synthesis of IAA, 1-aminocyclopropane-1-carboxylate deaminase, and siderophore.	[78]

**Table 2:** Summary of the positive effects of N, P, K, S, Mg, Ca and Si fertilization in mitigating abiotic stress conditions in Brassicas (2004–2020).

Crops	Abiotic Stresses	Macronutrients and Si Positive Effects	Reference
Radish	Zn toxicity	Mg^2+^ enhanced uptake and translocation of Zn, as well as alleviated Zn toxicity.	[164]
Mustard	Salinity	Nitrogen maintains synthesis of proline and ethylene to combat drastic impacts of salinity on photosystem.	[94]
	Cd toxicity	Nutrient solution of Mg fertilization improved shoot growth of *B. rapa* under cadmium (Cd) toxicity.	[145]
	Salinity	Nitrogen ameliorates salinity effects by improving growth attributes, physio-biochemical attributes (total chlorophyll, water content, stomatal conductance, K / Na ratio, carbonic anhydrase activity and malondialdehyde) and yield attributes (seeds pod^−1^, pod and yield plant^−1^).	[165]
	Cd toxicity	Silicon increased photosynthetic pigments and reduced inhibitory effects of Cd on root elongation.	[166]
	Salinity	Higher proline accumulation and photosynthetic efficiency increased plant growth with S fertilization.	[167]
	Heavy metals	Cadmium and lead have negative effects on P, Ca, Mn and Fe content root and leaves dry mass.	[168]
	Heavy metals	Application of Ca increases tolerance to Cd in mustard plants by restoring morphological and biochemical attributes.	[137]
	As toxicity	Silicon modulated root elongation with development of both primary and lateral roots.	[169]
	Cr toxicity	Silicon reduced transportation of Cr from root to shoot and photosynthetic activity by increasing net photosynthetic rate, chlorophyll, and carotenoid content.	[170]
	Salinity	Silicon increased plant growth, antioxidant activity (catalase, peroxidase and superoxide dismutase) and proline content.	[171]
	Heavy metals	Application of S mediated antioxidant enzymes in the plant, contributing to phytoextraction of potentially toxic elements (cadmium and zinc) from contaminated soils, helping in phytoremediation process of the soil.	[172]
Rapeseed	Drought	Potassium fertilization improved relative water content, stomatal conductance, relative chlorophyll index, and productivity.	[173]
	Salinity	Silicon nutrition ameliorated the lethal impacts of salinization in canola by lowering Na absorption, maintaining root cell integrity, reduced lipid peroxidation, enhancing the scavenging capability of ROS and decreased lignification.	[174]
	Drought	Fertilization with K_2_SO_4_ alleviated deleterious effects of water stress by stimulating productive characteristics (pods plant^−1^, seeds pod^−1^ and grain yield).	[130]
	Drought	Nitrogen improved proline production to maintain water balance and integrity of proteins, enzymes and cell membranes.	[107]
	Oxidative stress	Nitrogen and S reducing reactive oxygen species production.	[175]
	Salinity	Silicon application prevented toxic ions (Na and Cl) accumulation while maintaining K, P and Fe content in plants.	[176]
	Drought	Silicon increased shoot–root biomass, total chlorophyll content, activity of superoxide dismutase and catalase while reducing lipid peroxidation.	[177]
	Salinity	Phosphate fertilizers improved plant performance under salt stress by lowering Na^+^/K^+^ ratio and increasing P uptake.	[114]
	Drought	Nitrogen increased plant height, number of branches, number of fruits per plant, thousand seed weights and crude protein.	[178]
	Cd toxicity	Silicon reduced oxidative damage in plants by increasing antioxidant components and methylglyoxal detoxification system that enhance tolerance to Cd stress.	[179]
	Drought	Silicon improved antioxidants enzymes, ascorbate and glutathione pool, glyoxalase systems and proline by increasing protective role and maintaining redox status of plants.	[126]
	Oxidative stress	Silicon improved biomass, N uptake and chlorophyll content. Also, decreased oxidative stress by reducing hydrogen peroxide and malondialdehyde production.	[180]
	Haze condition	Nitrogen increased shoot biomass and photosynthetic productivity.	[181]
Canola	Salinity	Calcium-fortified composted animal manure alleviate oxidative stress, improvement in growth, physiology and mineral nutrition	[182]
	Salinity	Increased activity of phosphatase enzymes and reduced phosphate levels in plants.	[183]
	Salinity	Potassium fertilization mitigates the effects of salinity by confining Na absorption, activating cellular compartmentalization of excess Na^+^ in cell vacuole.	[129]
	Heavy metals	Sulfur application increases lipid peroxidation and activities of antioxidant enzymes.	[184]
	Drought	Fertilization of Ca allows plants to resist drought by improving antioxidant capacity, oil quality and essential fatty acids (linolenic acid and linolenic acid) in seeds.	[185]
	Drought	Potassium mitigated the effect of water deficiency by increasing water and nitrogen use efficiency, improving chlorophyll index, leaf area index, cell membrane integrity and productivity.	[186]
Cabbage	Drought	Nitrogen increased harvest index and dry matter production.	[187]
	Cd toxicity	Silicon alleviated Cd toxicity by increasing activities of antioxidant enzymes and shoot and root biomass.	[188]
	Salinity	Potassium fertilization improved absorption of total soluble free amino acids and proteins, proline content, regulated activities of antioxidant and improved gas exchange traits.	[189]
Broccoli	Heavy metals	Calcium fertilization mitigates ZnSO_4_ toxicity by increasing total phenolic content and antioxidant capacity of sprouts.	[190]
	Salinity	Nitrogen increased photosynthetic capacity and vitamin C content.	[191]
	NH_4_^+^ toxicity	Silicon alleviated NH_4_^+^ toxicity in cauliflower by increasing physical integrity of membranes while increasing water use efficiency in broccoli.	[192]
	Drought	Co-application of macro- and micronutrient and biostimulants increased nutritional status of broccoli plants in water deficient conditions.	[193]
	High luminosity	A positive correlation between Fe, Mg and Ca, and high light was observed.	[194]
Arugula	Drought	Potassium mitigated the effect of water deficiency by increasing water and nitrogen use efficiency, improving chlorophyll index, leaf area index, cell membrane integrity and productivity.	[195]
	Drought	Silicon improved gas exchanges capacity.	[196]
Kale	Drought	Silicon reduced water loss, increased shoot biomass and plant height.	[197]
Turnip	Heavy metals	Polypeptide Ca has a dual function in competitive inhibition in cadmium-contaminated agricultural land.	[198]

**Table 3:** Summary of the positive effects of micronutrients fertilization in mitigating unfavorable abiotic stress conditions in Brassicas (2005–2020).

Crops	Abiotic Stresses	Micronutrients Effects	Reference
Rapeseed	Cold temperature and high light radiation	Boron removal, mobilization and partitioning into root–shoot and younger leaves of plant were imperatively reduced in chilling temperature and intensive light.	[250]
	Drought	Drought drastically reduced Zn contents and led to photosynthetic damages with alternative reduction in transpiration, net assimilation and stomatal conductance and act as Cu-Zn SOD enzyme.	[251]
	Salinity	Protein and micronutrients were improved in order of Mn > Zn > Cu > Fe in aerial parts with application of N and Zn.	[252]
	Waterlogging	Waterlogging severely impaired growth and nutrients accumulation and ATP synthesis in plants.	[253]
	Waterlogging	Early waterlogging stress resulted in higher accumulation of Mn, Fe, Zn and Cu in the leaves and causes toxicity.	[243]
Turnip	Drought	Boron deficiency is increased under drought stress and led to the disturbance of electron transportation in photosynthesis, lowering chlorophyll content and root–shoot ratio.	[228]
	Drought	Drought drastically reduced Zn contents and led to photosynthetic damages with alternative reduction in transpiration, net assimilation and stomatal conductance.	[226]
Canola	Salinity	Micronutrients (Fe, Mn and Cu) contents in plant aerial parts were improved under salt stress	[254]
	Drought	Boron deficiency is increased under drought stress and led to the disturbance of electron transportation in photosynthesis, lowering chlorophyll content and root–shoot ratio.	[229]
	Drought	Yield and yield components were improved with lower dose of foliar Fe and Mn.	[231]
Mustard	Heavy metals	Accumulate high amount of Cd, Cu, Ni, Cr, Zn, Fe, Co, Pb and Se from contaminated sites.	[241]
	Heavy metals	High levels of Cd decrease micronutrients (Mn, Fe, Cu and Zn) content.	[255]
Broccoli	High light radiation	Short duration of blue light-emitting diodes (LED) prominently improves phytochemical components, essential micronutrient (B, Fe, Zn, Mn, Mo, Na and Cu) and macronutrients (Ca, P, K, Mg and S).	[248]
	Salinity	Salinity reduced yield and boron accumulation in aerial parts of plant.	[256]
	Hight light radiation	Short duration blue light enhanced different phytochemical activities, micronutrients (Zn, Mn, Mo, B, Na, Fe and Cu) concentration and also macronutrients (Ca, P, K, S and Mg) concentration in plants.	[248,249]
	Salinity	Biofertilizers improve Fe availability and also Ca and Mg content in plants.	[257]
Chinese cabbage	Cold temperature	Molybdenum promotes antioxidant and phytochemical activities, improve growth, quality and yield.	[258]
*A. thaliana*	High light radiation	Zinc prevents photo-inhibitory damages to photosynthetic apparatus by producing ROS and enhancing carotenoid contents plant leaves.	[259]

## Data Availability

No new data were created or analyzed in this study. Data availability is not applicable to this article.
